# Significant reduction of vancomycin resistant *E*. *faecium* in the Norwegian broiler population coincided with measures taken by the broiler industry to reduce antimicrobial resistant bacteria

**DOI:** 10.1371/journal.pone.0226101

**Published:** 2019-12-12

**Authors:** Roger Simm, Jannice Schau Slettemeås, Madelaine Norström, Katharine R. Dean, Magne Kaldhusdal, Anne Margrete Urdahl

**Affiliations:** 1 Institute of Oral Biology, University of Oslo, Oslo, Norway; 2 Norwegian Veterinary Institute, Oslo, Norway; Ross University School of Veterinary Medicine, SAINT KITTS AND NEVIS

## Abstract

Vancomycin resistant enterococci (VRE) belong to the most common causes of nosocomial infections worldwide. It has been reported that use of the glycopeptide growth promoter avoparcin selected for a significant livestock-reservoir of VRE in many European countries, including Norway. However, although avoparcin was banned as a feed-additive in 1995, VRE have for unknown reasons consistently been reported in samples from Norwegian broilers. When avoparcin was banned, broiler-feed was supplemented with the polyether ionophore narasin in order to control the diseases coccidiosis and the frequent sequela necrotic enteritis. A potential link between transferrable vancomycin resistance and reduced susceptibility to narasin was recently reported. The use of narasin as a feed additive was abolished by the Norwegian broiler industry in 2016 and since then, broilers have been reared without in-feed antibacterial supplements. In this study, we demonstrate that all VRE isolates from Norwegian broilers collected in 2006–2014 displayed reduced susceptibility to narasin. Surveillance data collected two years after the narasin abolishment show a significant reduction in VRE, below the detection limit of the surveillance method, and a concurrent marked reduction in *Enterococcus faecium* with reduced susceptibility to narasin. The significant decline of *E*. *faecium* with reduced susceptibility to these antimicrobial compounds also coincided with an increased focus on cleaning and disinfection between broiler flocks. Furthermore, data from a controlled *in vivo* experiment using Ross 308 broilers indicate that the proportion of *E*. *faecium* with reduced susceptibility to narasin was heavily reduced in broilers fed a narasin-free diet compared to a diet supplemented with narasin. Our results are consistent with that the abolishment of this feed additive, possibly in combination with the increased focus on cleaning and disinfection, has had a substantial impact on the occurrence of VRE in the Norwegian broiler population.

## Introduction

Enterococci are part of the intestinal microbiota of humans and animals, but they are also a major cause of nosocomial infections worldwide [1]. An important contributing factor to the success of these opportunistic pathogens is the prevalent resistance to many antimicrobial compounds. The increasing spread of vancomycin resistance among enterococci is especially worrisome, since vancomycin is regarded as a drug of last resort for the treatment of multi-drug resistant Gram-positive infections in humans, and the drug of choice for treatment of methicillin resistant *Staphylococcus aureus* (MRSA) [2]. Although the strains of enterococci causing the majority of hospital acquired infections may differ from the strains most often isolated from animals [3, 4], genetically related vancomycin resistant enterococci (VRE) have been shown to colonize both animals and humans [3, 5, 6]. Transfer of vancomycin resistance can occur between isolates of animal origin and human clinical isolates, both *in vitro* and *in vivo* in humans and animals [7–9]. In addition, interspecies transfer of vancomycin resistance between enterococci and other Gram-positive bacteria including staphylococci can occur [1, 10]. There is therefore a concern regarding community reservoirs of VRE that can transfer to humans and complicate treatment of infections.

Use of the glycopeptide avoparcin as a feed additive selected for a substantial population of VRE in livestock in many European countries [11–14]. Because of this, the use of avoparcin as a feed additive was banned in Norway in 1995 and in the European Union in 1997. Following the ban, many countries reported reduced occurrence of VRE in livestock [15–18]. However, even after decades of glycopeptide-free feed, a reservoir of VRE still exists in food producing animals in Europe. In Norway, surveillance of VRE in animals, feed and food products fall under the mandate of NORM-VET, the Norwegian monitoring program for antimicrobial resistance in the animal and food production sectors. During the years surveillance of VRE has been performed by NORM-VET, it has consistently been detected in samples from broilers [19–25]. The reason for the occurrence of a persistent reservoir of VRE in Norwegian broiler production is currently unknown. Avoparcin was introduced as a feed additive in chicken feed in 1987 in an attempt to prevent epidemics of necrotic enteritis in the Norwegian broiler population and this measure successfully kept the disease under control in the following years. When avoparcin was banned in 1995, necrotic enteritis reemerged. In response to this, the coccidiostat narasin was introduced as a feed additive in broiler feed [26]. Narasin is a polyether ionophore with anticoccidial as well as antibacterial properties [27]. Introduction of narasin resulted in a gradual decline in the prevalence of clinical necrotic enteritis, which has remained low since 1998 [26, 28]. However, due to intensive negative publicity in Norwegian mass media regarding the use of narasin as a feed additive, major retail chains wished to offer their customers broilers reared without in-feed coccidiostats. The stakeholders of Norwegian broiler production thereby started phasing out narasin from broiler feed in 2015, and since 2016, all broilers in Norway has been raised without routine use of antibacterial feed additives. Anticoccidial vaccines were introduced to control parasite infections and so far, since the discontinuation of in-feed coccidiostats, occurrence of clinical coccidiosis or necrotic enteritis has been of no major concern.

A potential link between vancomycin resistance and reduced susceptibility to narasin has been discussed for many years, and Nilsson *et al*. recently reported experimental evidence supporting this hypothesis [9, 29]. They observed occasional co-transfer of reduced narasin susceptibility together with vancomycin resistance during conjugation experiments between *E*. *faecium* strains [9]. Sequencing of the transferred plasmids indicated that a region consisting of a two-gene operon encoding a putative ABC-type membrane transporter was associated with decreased susceptibility to narasin [29].

Following the discontinuation of narasin as a feed additive, Norwegian broiler production provides a unique opportunity to study the potential role of narasin in upholding a reservoir of VRE in the broiler population. This study summarizes the surveillance data collected for VRE and *E*. *faecium* with reduced susceptibility to narasin in the NORM-VET program in the years 2000–2018 and reports molecular detection of potential resistance mechanisms, confirming a strong correlation between vancomycin resistance and reduced susceptibility to narasin. We report a substantial reduction in *E*. *faecium* isolates with reduced susceptibility to narasin in samples from broilers and discuss these data in light of a controlled *in vivo* experiment showing correlation between in-feed narasin and occurrence of *E*. *faecium* with reduced susceptibility to narasin. VRE were not detected in samples from Norwegian broilers in 2018 using a directly selective method screening for vancomycin resistance. The role of narasin in maintaining a VRE-reservoir is discussed as well as potential alternative explanations for VRE being below the detection limit of our method, in samples from Norwegian broilers.

## Materials and methods

### VRE and *E*. *faecium* with reduced susceptibility to narasin in Norwegian broilers

#### Surveillance data

Data on *E*. *faecium* from broilers collected in the NORM-VET program from 2002 to 2018 [19–25, 30–41], were extracted from the internal recording system of the Norwegian Veterinary Institute. The surveillance is performed on national level, and designed to ensure representative sampling. Data management was performed in R version 3.5.3 (RCoreTeam, 2019) and in SAS SAS-PC system version 9.4 for Windows (SAS Institute inc., Cary, NC, USA).

#### Isolates from the NORM-VET surveillance program

The isolation and identification of indicator *E*. *faecium* in the NORM-VET program follows standard guidelines used in general bacteriology. In short, faecal, caecal or boot swab samples are plated directly on Slanetz and Bartley agar (Oxoid, Thermo Fisher Scientific Inc., Waltham, Massachusetts, USA). A single colony is randomly selected, streaked on blood agar, identified and subsequently susceptibility tested against a panel of antibiotics. For meat samples, plating on Slanetz and Bartley agar occur after enrichment in Azide dextrose broth (Oxoid). These methods are referred to as the traditional methods in the current study. In addition, directly selective methods to isolate VRE have been used instead of or in parallel with the traditional methods. In these methods that we refer to as the selective methods in the current study, samples are directly plated on Slanetz and Bartley agar containing 32 mg/L vancomycin before 2014 and 4 mg/L vancomycin from 2014.

Species confirmation was done by negative catalase reaction (all years), rapid ID32 STREP test (bioMérieux, Marcy l’Etoile, France) (1999 and 2000), ddlID PCR [42] (from 1999 to 2014) or by use of a matrix-assisted laser desorption/ionization time of flight apparatus (MALDI-TOF Microflex, Bruker Daltonik GmbH, Bremen, Germany) (from 2014). Minimum inhibitory concentration (MIC) values were obtained by using a broth microdilution method. All isolates collected between 2000 and 2013 were susceptibility tested with VetMIC^™^ panels (Dep. of Antibiotics, National Veterinary Institute, Sweden), while isolates from 2014 to 2018 were tested with Sensititre^®^ panels (TREK Diagnostics, LTD). Isolates were classified as vancomycin resistant if the MIC values were above the epidemiological cutoff (ECOFF) value 4 mg/L, as defined by the European Committee on Antimicrobial Susceptibility Testing (EUCAST, www.eucast.org). Detailed methodology and information on samples can be found in the NORM-VET reports [21–25, 35, 37, 41], and summarized in S1 Table.

All VRE isolates from broilers, isolated by the selective methods, and retrieved through the NORM-VET surveillance program in the years 2006–2018 were included in the present study (77 isolates). In addition, all *E*. *faecium* isolates collected from broilers during 2014 were included to screen for narasin susceptibility.

#### Narasin susceptibility testing

Susceptibility testing against narasin was included in the antimicrobial resistance analyses performed in NORM-VET from 2002–2011, and in 2018. In the current study, all *E*. *faecium* from broilers collected in 2014 were tested against narasin to complete the data set. The 2014 *E*. *faecium* were tested for narasin susceptibility using custom-made VetMIC^™^ plates containing only narasin. The test range in the VetMIC^™^ narasin plates was 0.06–64 mg/L and with the possibility to test up to eight isolates per plate.

#### Molecular detection of *vanA* and a putative narasin resistance mechanisms

DNA was extracted from all 77 VRE isolated between 2006 and 2014 by NUCLISENS^®^ easyMAG^®^ (bioMérieux) according to the manufacturers description. Presence of putative resistance genes was analyzed using HotStarTaq polymerase (QIAGEN, Hilden, Germany) in 25 μl PCR reactions containing HotStarTaq Master mix, 0.2 μM of each primer and 2 μl DNA template. The type of vancomycin resistance was determined using a multiplex PCR with the primers VanA.F (CATGAATAGAATAAAAGTTGCAATA) and VanA.R (CCCCTTTAACGCTAATACGATCAA) testing for *vanA* and VanB.F (GTGACAAACCGGAGGCGAGGA) and VanB.R (CCGCCATCCTCCTGCAAAAAA) detecting *vanB* [43]. Presence of the genes of the putative narasin resistance mechanism was analyzed with the primer pairs: ABCpermease_For (AGCTGCGTATGGCTCCATTT) and ABCpermease_Rev: (GCTGATGCTAAGCCAATGCC) detecting the genes encoding the permease and ABC_ATPase_For (TGTTCCTGGGGATGTTGCTC) and ABC_ATPase_Rev (AGAGCGTCGCAAGTTTCTCA) detecting the genes encoding the ATPase subunits [29]. PCRs were run at 94 °C for 15 min, 35 cycles of (94 °C, 20 s; 58 °C (*vanA*/*vanB*) or 63 °C (ABC-genes); 30 s; 72 °C, 50 s), and 72 °C, 10 min.

#### Statistical analyses

Data analysis was performed in SAS-PC System^®^ v 9.4 for Windows (SAS Institute Inc., Cary, NC, USA) and in R version 3.5.3 Copyright (C) 2019 The R Foundation for Statistical Computing Platform. The 95% confidence intervals were calculated by the exact binomial test using R version 3.5.3 for Windows (R Development Core Team, 2019). The Chi-squared Test for Trend in Proportions was used to assess an increasing or decreasing trend over time. The z-test was used to compare two proportions in 2018 compared to 2014. To determine if there was a difference in the narasin MIC values between *E*. *faecium* (traditional) and VRE (selective), we used a permutation test of independence implemented with the coin (coin_1.3–1) library.

### *In vivo* experiment testing the effect of narasin on the *E*. *faecium* population

#### Animals & housing

*In vivo* experiments were carried out at Scandinavian Poultry Research in Våler, Hedmark, Norway, using day-old Ross 308 broilers obtained from a commercial hatchery (Nortura Samvirkekylling, Våler, Norway). The broilers were housed in floor pens of 5.6 m^2^ on new wood shavings in a climate controlled poultry research facility. Water and pelleted feed were given *ad libitum*. All birds were challenged with a mixture of five different live attenuated strains of intestinal coccidia (a ten-fold dose of the vaccine Paracox^®^-5 (MSD Animal Health, Netherlands) on day 18. No other vaccines were administered throughout the study. Experiments were conducted in agreement with the Norwegian Regulation of Animal Experimentation, and approved by the national competent authority (Norwegian Food Safety Authority, FOTS ID 8179).

#### Experimental design

A total of 1760 day-old Ross 308 broilers were randomly allocated into two treatment groups, each group comprising eleven replicate pens with 80 broilers per pen. The setup was performed three times (with a total of 5280 birds). All experiments included a treatment group offered starter and grower feeds supplemented with 70 mg/kg of the polyether ionophore and coccidiostat narasin (Monteban^®^, Elanco Animal Health, USA), and a treatment group receiving the same basal feeds without antimicrobial feed additives.

#### Sampling for enterococci

Samples of caecal content were collected from 22, 23 or 24 days old broilers that were randomly selected and terminated by stunning the bird with a blow to the head followed immediately by cervical dislocation.

In total 198 caecal samples from broilers were analyzed for presence of VRE; 99 from broilers fed a diet supplemented with narasin and 99 from broilers fed a diet without antimicrobial feed additives. Samples were collected from three independent experiments. One hundred mg of caecal material was dissolved in 1 mL PBS and the suspension was plated onto a Slanetz and Bartley agar plate with vancomycin (4 mg/L). The agar plates were incubated at 37 °C for 48h and growth of bacteria was observed.

In parallel, a total of 125 caecal samples were used to analyze the proportion of bacteria with reduced susceptibility to narasin in the *E*. *faecium* population; 60 from broilers fed a diet supplemented with narasin and 65 from broilers fed a diet without narasin.

The samples were ten-fold serially diluted and plated on Slanetz and Bartley agar with vancomycin (4 mg/L) or narasin (2 mg/L) and without antimicrobial supplement. The bacteria were incubated at 37 °C for 48h and the number of colony forming units (CFU) were counted. Bacteria from the different colony morphologies were streaked on blood agar plates and the species were determined by MALDI-TOF analysis (Bruker Daltonics). *E*. *faecium* consistently showed up as red or pink colonies with a diameter of 1–2 mm, the phenotype was lighter in color on narasin-supplemented agar. Other species detected were *Enterococcus hirae* and rarely *Lactobacillus johnsonii*, but these species generally displayed smaller colony morphologies. Based on these initial experiments, red and pink colonies with a diameter of 1–2 mm were counted as *E*. *faecium* and random testing of colonies confirmed this presumption. The total number of CFU corresponding to *E*. *faecium* on each plate was determined and the ratio of vancomycin resistant bacteria or bacteria with reduced susceptibility to narasin and the total number of bacteria (resistant and susceptible) was calculated for each sample. The detection limit for this experiment was 1000 bacteria per mg caecal matter.

The average values and 95% confidence intervals were calculated for each population and statistical significance was calculated using the two tailed Students t-test, with unequal variance.

## Results

### Surveillance data show a reduction of VRE in Norwegian broilers

**Fig 1:**
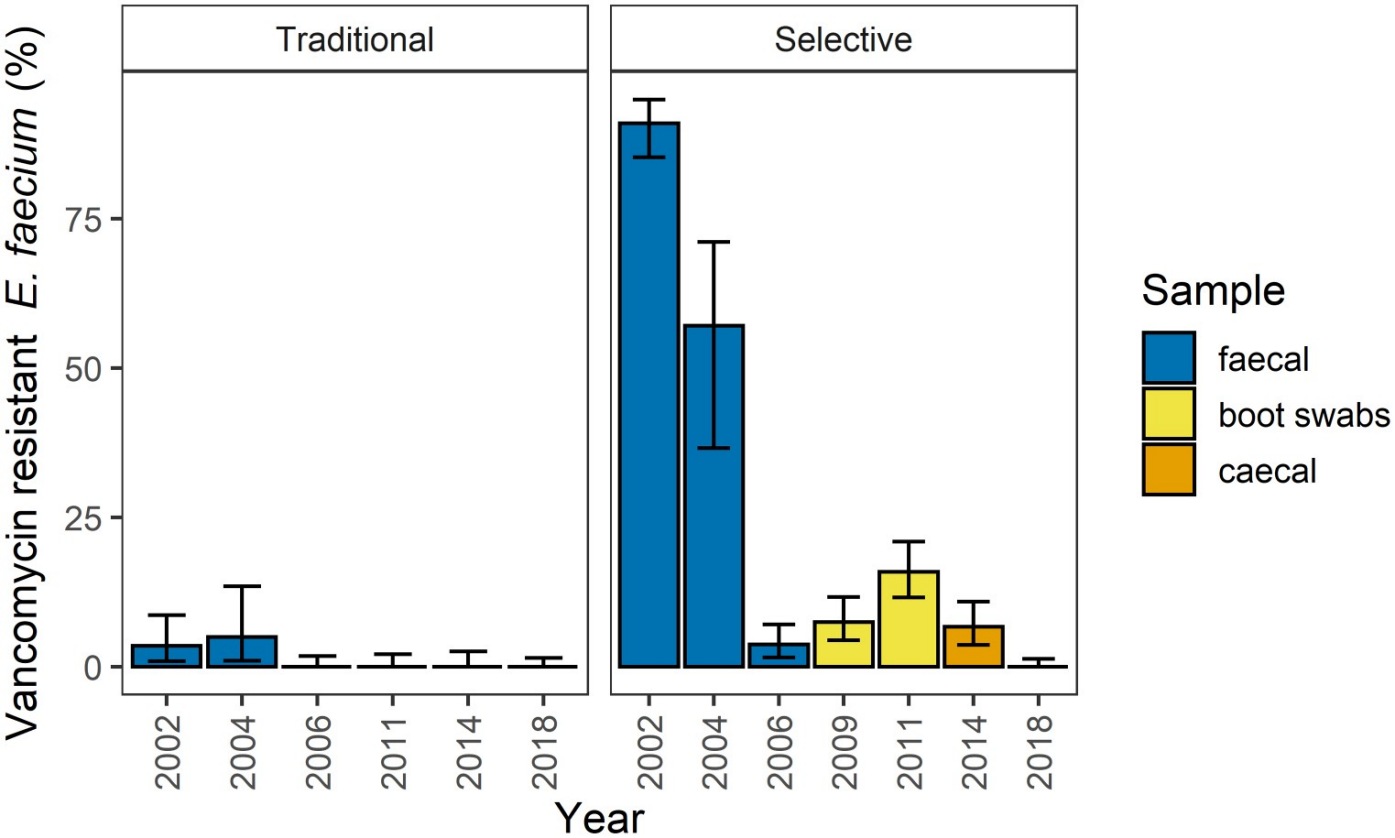
Occurrence of vancomycin resistance among *E*. *faecium* isolated by the traditional methods in broiler flocks from 2002 to 2018 (traditional) and prevalence of VRE in broiler flocks isolated by the selective methods in the same time period (selective). The total number of isolates (N) tested by the traditional methods were 115 (in 2002), 62 (2004), 200 (2006), 0 (2009), 176 (2011), 143 (2014) and 251 (2018). The number of samples tested with the selective methods were 155 (in 2002), 35 (2004), 219 (2006), 228 (2009), 252 (2011), 210 (2014) and 280 (2018). Shown are the relative numbers of vancomycin resistant isolates in percent. Error bars correspond to 95% confidence intervals.

Vancomycin resistance was detected in 3.5%, 5% and 0% of the *E*. *faecium* isolated by the traditional method from faeces in 2002, 2004, and 2006, respectively (Fig 1; S2 Table). This indicates a decreasing trend of vancomycin resistance among *E*. *faecium* from broiler flocks in the first years of surveillance (χ^2^ = 5.8 p = 0.02). Since then, i.e. in the years 2011, 2014 and 2018, none of the *E*. *faecium* isolates collected with the traditional methods have shown reduced susceptibility to vancomycin (Fig 1; S2 Table).

The more sensitive selective methods, which identifies VRE directly in the samples, detected VRE in 91%, 57.2%, and 2.3% of faecal samples collected in 2002, 2004 and 2006, respectively (Fig 1; S2 Table). These results show a clearly declining trend in prevalence of VRE (χ^2^ = 288, df = 1, p-value < 2.2 x10^-16^) and support the data from the traditional methods. In the following sampling periods, VRE were detected by the selective methods in 7.5% of boot swab samples in 2009, and 15.9% in 2011. In 2014, 6.7% of caecal samples contained VRE, whereas none of the caecal samples contained VRE in 2018 (Fig 1; S2 Table), which represents a significant decrease (p = 9.1 x 10^−14^) from 2014. This is the first time since the introduction of the selective methods, that no VRE were detected.

### Significant reduction of *E*. *faecium* with reduced susceptibility to narasin in broilers

**Fig 2:**
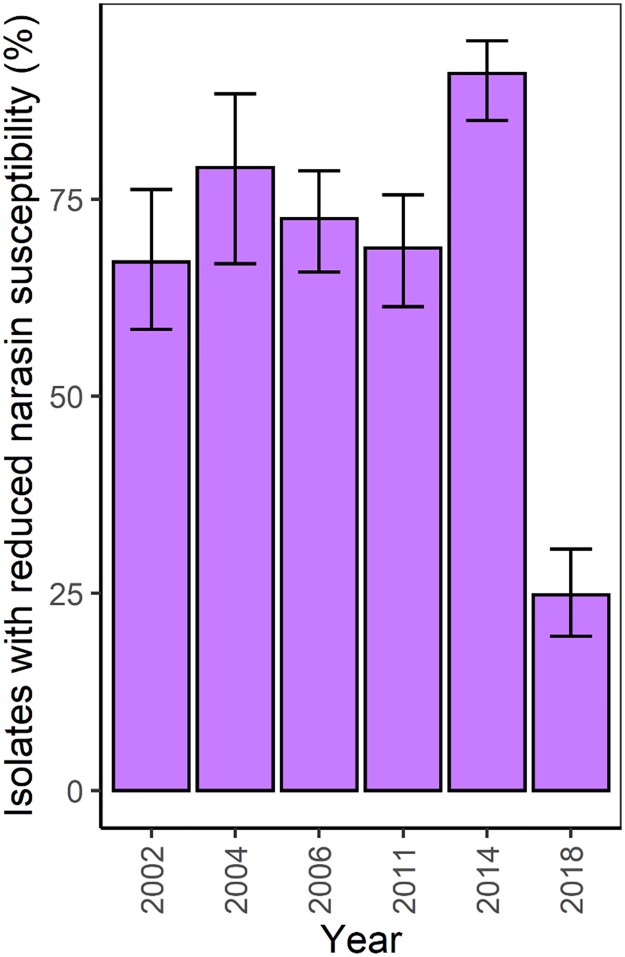
Prevalence of *E*. *faecium* with reduced susceptibility to narasin isolated from Norwegian broiler flocks in NORM-VET during the years 2002–2018. The number of isolates during the years were 115, 62, 200, 176, 143 and 250, in 2002, 2004, 2006, 2011, 2014 and 2018, respectively. Error bars indicate 95% confidence intervals.

All *E*. *faecium* from broilers collected in 2014 were susceptibility tested against narasin, and these results were compiled with the previously accumulated surveillance data for comparison (Fig 2). The proportion of the *E*. *faecium* population that has had a narasin MIC > 2 mg/L has varied between 69% and 91%, from 2006–2014, displaying the highest value in 2014 (Fig 2). In 2018, there was a significant reduction in the occurrence of *E*. *faecium* with reduced susceptibility to narasin to 24.7% (p = 3.4 x 10^−19^) as compared to the data observed in 2014.

### Strong association between vancomycin resistance and reduced susceptibility to narasin among *E*. *faecium* in samples from Norwegian broilers

**Fig 3:**
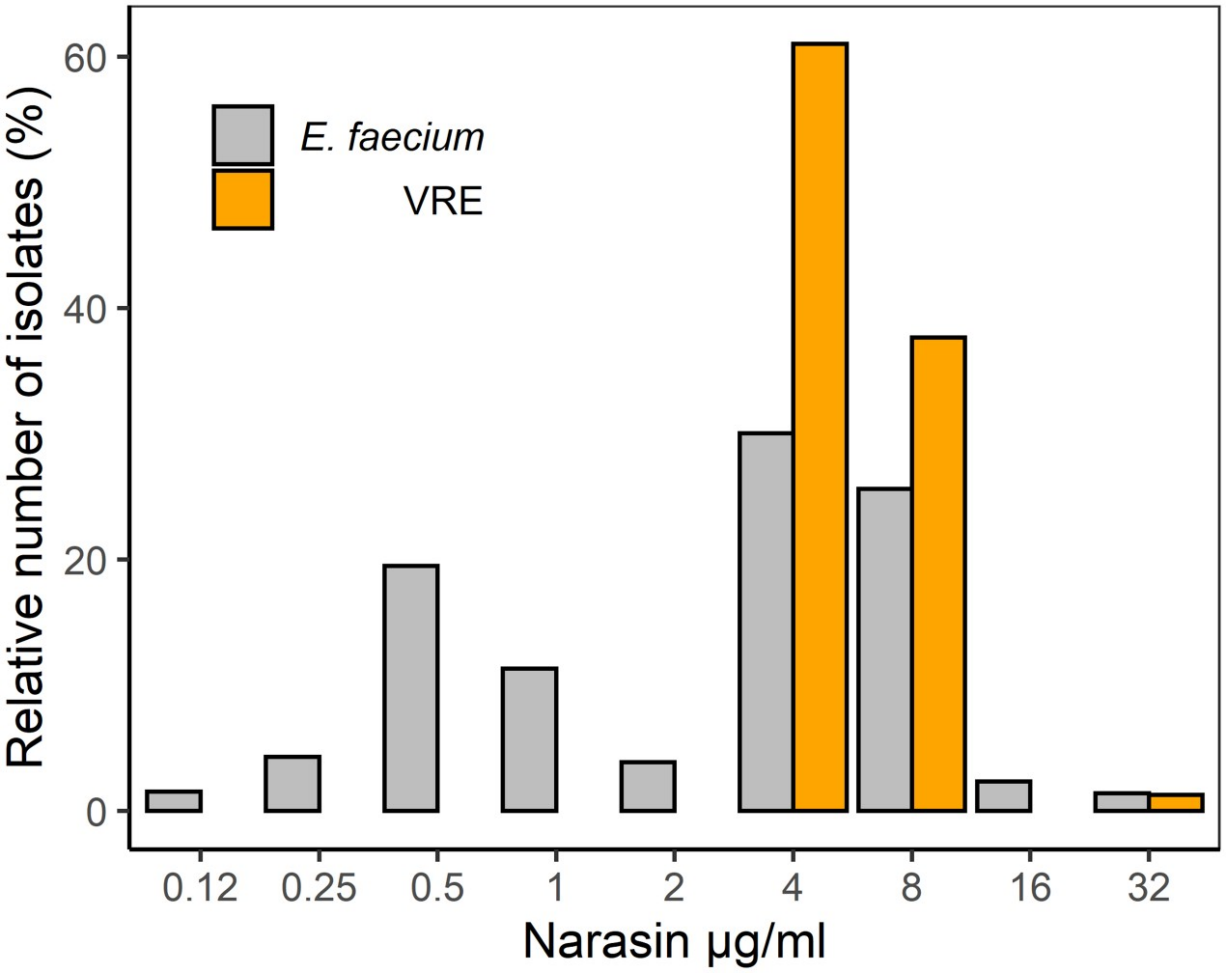
Distribution of MIC values of narasin among *E*. *faecium* isolated by the traditional methods (traditional; N = 769) and VRE isolated by the selective methods (selective; N = 77) between the years 2006 and 2018.

The narasin susceptibility of the *E*. *faecium* isolates collected between 2002 and 2018 varied, with MIC values ranging from 0.125–32 mg/L (S2 Table). The MIC values display a tendency toward a bimodal distribution with an ECOFF value of 1–2 mg/L (Fig 3), as previously suggested [29]. All the 77 VRE isolates displayed reduced susceptibility to narasin, with MIC values of 4–8 mg/L (Fig 3). We found that there was a significant difference (p-value = 2.521e^-8^) among the narasin MIC values between *E*. *faecium* in general and VRE.

PCR analyses demonstrated that all VRE isolates were positive for *vanA*, and for the putative narasin resistance genes (S1 Appendix).

### *In vivo* experiments demonstrate that in-feed narasin selects for *E*. *faecium* with reduced susceptibility to narasin

**Fig 4:**
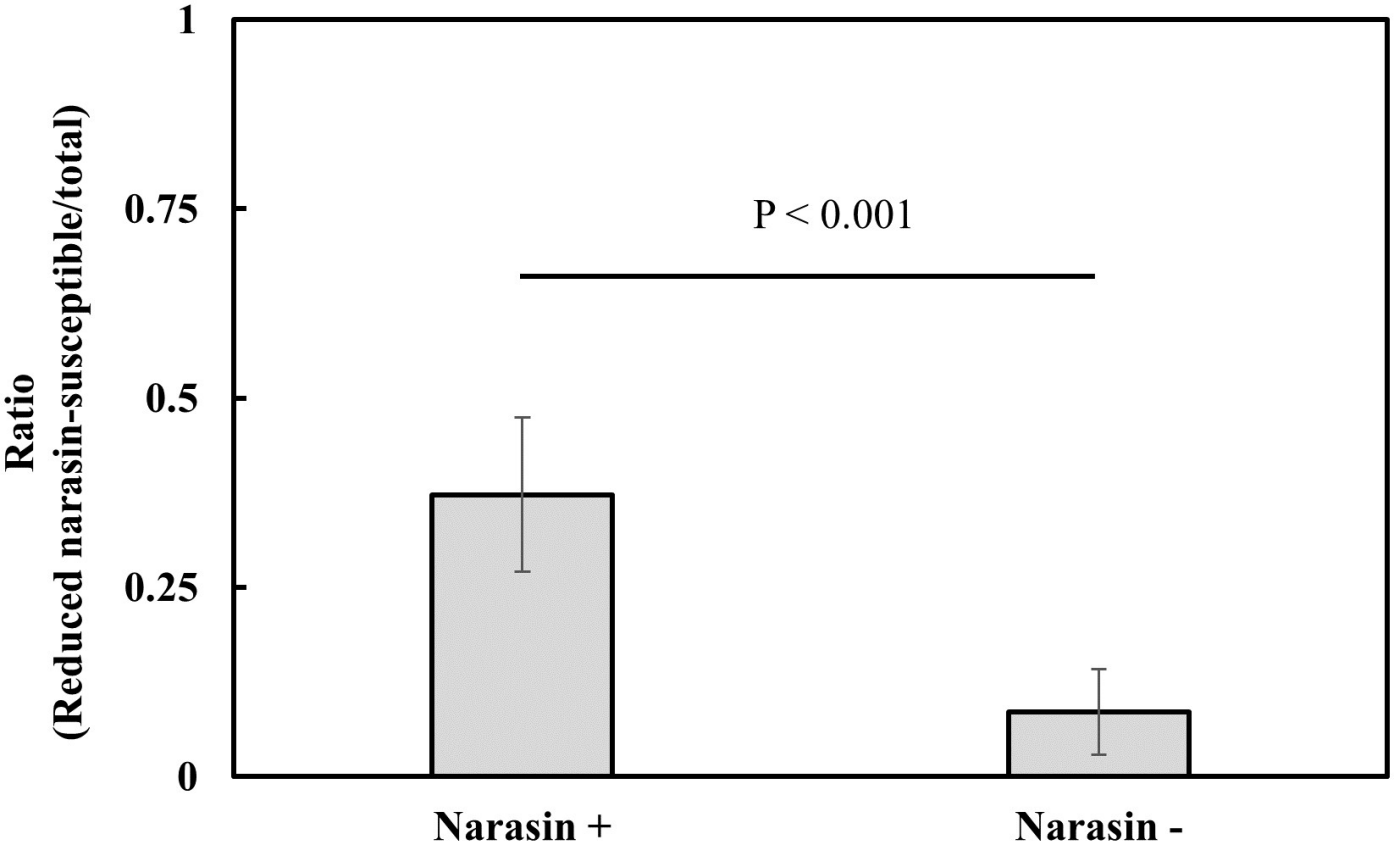
The proportion of *E*. *faecium* with reduced susceptibility to narasin is higher in broilers fed diets with narasin than those fed a narasin-free diet. Broilers were fed a diet with (Narasin +; N = 60) or without (Narasin -; N = 65) supplementation with narasin (70 mg/Kg). The number of *E*. *faecium* with reduced susceptibility to narasin and the total number of *E*. *faecium* were determined. Shown are average values of the ratio of *E*. *faecium* with reduced susceptibility to narasin compared to the total number of *E*. *faecium*. Error bars represent 95% confidence intervals. Statistical significance was calculated using the two-tailed Student’s t-test with unequal variance.

To test the selective effect of in-feed narasin on the populations(s) of *E*. *faecium* that display reduced susceptibility to narasin and/or vancomycin resistance, a controlled animal experiment was performed. VRE were not detected in this experiment. The fraction of *E*. *faecium* with reduced susceptibility to narasin in caecal material was 4.3-fold higher in broilers fed a diet containing narasin compared to broilers fed a narasin-free diet (Fig 4; S1 Appendix).

## Discussion

To our knowledge, the Norwegian surveillance data from 2018 is the first report in which VRE were not detected in broilers, on a national basis in a country that previously used avoparcin as a feed additive, when the selective method was used for detection. The data summarized in this study together with previously published data, demonstrate that the prevalence of VRE in Norwegian broilers have been declining since the avoparcin ban in 1995. VRE were detected by a selective method in 97% of faecal samples from poultry (broilers and turkeys) in June 1995, the month directly following the ban of avoparcin [44]. The occurrence of VRE persisted at this level in faecal material from Norwegian poultry sampled between September 1995 –January 1997 [44]. In a follow-up study, VRE were detected in 99% of faecal samples from poultry collected during the summer of 1998, three years after the avoparcin ban [45]. However, between 1998 and 2003, Sørum *et al*. reported a declining trend of VRE in poultry samples, from 100% to 72% [46]. These data correlate with the surveillance data from NORM-VET [22–24], showing a steeply declining trend of VRE in broiler samples between 2002 and 2006 (Fig 1). Between 2006 and 2014, the prevalence of VRE remained relatively low and in 2018, the prevalence was below the limit of detection. However, comparison of data from year to year should be made with caution since the sampling method has changed, first in 2009 and then again in 2014 (S1 Table). The published data from 1995–2003 [44–46], and the NORM-VET data from 2000–2006 [22–25], represent samples from faeces. In 2009 and 2011, samples were from boot swabs, sampling an area of the floor of the broiler houses [21, 35] and in 2014 and 2018, samples were collected directly from the caecum of ten broilers per flock and pooled [37, 41].

The reason for the persistence of VRE in Norwegian broilers, for at least 19 years, after the avoparcin ban is not completely understood, and neither is the reason for the absence of VRE in samples from 2018. It has previously been suggested that plasmid maintenance systems and transmission by horizontal gene transfer in combination with unknown selection pressure (including host factors) may contribute to the persistence of VRE on poultry farms [47–49]. No host factors directly promoting persistence of VRE in poultry has been identified, however horizontal transfer of a VanA-encoding plasmid was increased in a mouse model compared to *in vitro* models [7]. The ω-ε-ζ toxin-antitoxin system that is often found on VanA-encoding plasmids from Norwegian poultry, can stably maintain the plasmid in a population of *E*. *faecium in vitro* and *in vivo* [47, 49]. In addition, the axe-txe toxin-antitoxin system has been associated with clinical and livestock VRE-isolates. However, there are VRE-isolates from broilers in which neither the ω-ε-ζ, nor the axe-txe systems have been detected [48] suggesting that this type of plasmid maintenance systems cannot be the sole explanation.

The significant reduction of VRE between 2014 and 2018 coincided with the abolishment of narasin as a feed-additive in Norwegian broiler production in 2016. Rearing broilers on narasin-free feed has had a substantial effect on the subpopulation of *E*. *faecium* with reduced susceptibility to narasin, reducing it from 91% in 2014 to 24.7% in 2018 (Fig 2). The data from our animal experiment display a similar approximately four-fold difference between broilers receiving narasin-free and narasin-containing diets. The strong selective pressure by in-feed narasin can also be inferred from surveillance data showing very low occurrence of *E*. *faecium* with reduced susceptibility to narasin in animal species that do not receive narasin in the diet [34, 36, 50]. This includes layer-hens for egg production, that never received narasin in the diet, and in 2013 had a prevalence of *E*. *faecium* with reduced susceptibility to narasin of only 1.9% [36]. Although in-feed narasin has been suspected to contribute to the persistence of VRE in broilers, this hypothesis has usually been disregarded since no narasin resistance determinant was known. However, in light of the recent discovery of a putative narasin resistance mechanism that often is physically linked to the *vanA* gene cluster [9, 29], the Norwegian surveillance data support a role for narasin in maintaining vancomycin resistance in the *E*. *faecium* population of broilers. All VRE in the biobank of NORM-VET collected between 2006 and 2018 display reduced susceptibility to narasin, i.e. MIC > 2 mg/L. Similar results have been reported for isolates collected from intestinal content of broilers in Sweden [51, 52]. Our molecular analyses demonstrated the presence of *vanA*, and the genes encoding the ABC-type membrane transporter putatively conferring reduced susceptibility to narasin in all VRE isolates from Norwegian broilers. The VanA-encoding operon is situated on the non-conjugative Tn*1546* transposon, which can integrate into chromosomal and plasmid DNA and be transferred to susceptible bacteria by integration into transferrable plasmids [53]. The sequenced VanA-encoding plasmids from Norwegian and Swedish poultry display mosaic structures indicating multiple recombination events shaping the plasmids [29, 47, 49]. Considering that the majority of *E*. *faecium* in both Sweden and Norway (before the discontinuation of in feed-narasin) display reduced susceptibility to narasin, it is likely that persistence of the Tn*1546* element and the concomitant vancomycin resistance is, at least partly, mediated via integration of Tn*1546* on narasin resistance plasmids. In the years prior to the introduction of narasin as a feed additive, broilers were reared with a combination of a polyether ionophore (monensin, lasalocid or salinomycin) and avoparcin. This was followed by a brief period between the ban of avoparcin and the introduction of narasin where monensin only was used as a feed additive [26]. It is conceivable that the simultaneous use of a glycopeptide and a polyether ionophore has selected for chimeric plasmids encoding both vancomycin and narasin resistance mechanisms. It is also possible that this created strains with separate DNA molecules encoding the vancomycin and narasin resistance mechanisms. In either case, the subsequent use of narasin conferred a selective pressure preserving these vancomycin resistant strains with reduced susceptibility to narasin in the *E*. *faecium* population. This contradicts the conclusions of a recent study by Nilsson *et al*. but is not incompatible with their findings. They show that in Swedish broilers, the VRE population has been declining from 2005 to 2015, in spite of continuous use of in-feed narasin and conclude that the role of narasin in persistence of VRE is questionable [54]. However, although horizontal gene transfer, plasmid maintenance systems and selective pressure exerted by narasin can explain the persistence of plasmid borne resistance mechanisms in a bacterial population, broiler production is based on an all-in, all-out strategy. In this approach, the broiler house is emptied of the entire broiler flock and the broiler house is cleaned and disinfected before the next broiler flock is introduced. This means that VRE have to be introduced into the broiler house with new flocks or persist in the broiler house or its immediate environment. If VRE were introduced with the broiler flocks, we expect to find VRE among breeder flocks. However, VRE were not detected in environmental samples from hatcheries in Norway in 1999 [55] or Sweden in 2004 [56]. This does not exclude that VRE may have been imported from the breeding stock and (re)introduced on broiler farms via hatchlings and that changes in production of breeding animals may have affected the occurrence of VRE in the Norwegian broiler population. However, it is more likely that the continuing occurrence of VRE is due to a persistent population of VRE in the broiler houses. This has previously been described in studies from several European countries, including Norway and Sweden [18, 55–57]. Given that all VRE display reduced susceptibility to narasin, this subpopulation would be included in the bacteria selected for by the use of in-feed narasin. Assuming that VRE are reintroduced into the broiler houses at a low frequency, the small subpopulation of persistent VRE with reduced susceptibility to narasin would be outcompeted over time by the much larger population of vancomycin susceptible *E*. *faecium* with reduced susceptibility to narasin. Hence, the proportion of VRE would decline over time, even in broilers fed narasin, correlating with the results of Nilsson *et al*. [54]. However, we hypothesize that the occurrence of VRE would have dropped below detection limit faster without the use of narasin as a feed additive and our results support this hypothesis.

In parallel with the withdrawal of narasin as a feed additive, the Norwegian poultry industry introduced a number of measures with the intention to reduce the occurrence of extended-spectrum cephalosporin (ESC) resistant *Escherichia coli*, including improved cleaning and disinfection routines. Mo *et al*. reported that the risk of detecting ESC-resistant *E*. *coli* in Norwegian broiler flocks was mainly associated with previous ESC-resistant flocks in the same broiler house, and failure to disinfect the floor of the broiler house between flocks [58]. This suggests that recirculation of antimicrobial resistant bacteria in the Norwegian poultry houses is a major contributor to persistent antimicrobial resistant bacterial populations [58]. Similar results have been reported previously and it has been demonstrated that VRE persist in the farm environment despite cleaning and disinfection between flocks [55, 56, 59, 60]. There is also the possibility that VRE can spread between farms via contaminated equipment, vehicles or people [56]. Improved disinfection routines have been shown to reduce the occurrence of VRE [61], and the increased focus on cleaning and disinfection of poultry farms implemented by the Norwegian poultry industry in the time between 2014 and 2018 may very well have contributed to VRE being below the detection limit of the surveillance methods in broilers in 2018. This is supported by the significant reduction of *E*. *coli* resistant to ESC in broilers between 2016 and 2018 [39, 41], but opposed by the slight increase in quinolone resistant *E*. *coli* in the same time period [39, 41].

## Conclusions

For the first time since the avoparcin ban in 1995, VRE were not detected in samples from Norwegian broilers. This was accompanied by a significant reduction of *E*. *faecium* with reduced susceptibility to narasin. The molecular mechanism conferring vancomycin resistance is closely associated with the putative narasin resistance mechanism, suggesting that the discontinuation of narasin as a feed additive contributed to the reduction of VRE. Also, improved cleaning and disinfection routines introduced in parallel with the withdrawal of narasin is likely to have contributed to the absence of VRE in broilers. To follow the situation, VRE in the Norwegian broiler population should continue to be monitored in the future.

## Supporting information

S1 Appendix (DOCX)

S1 Table Isolation methods. (DOCX)

S2 Table MIC values of isolates. (XLSX)

S3 Table CFU feed additive experiment. (XLSX)
