# Supplementary material for: Significant reduction of vancomycin resistant E. faecium in the Norwegian broiler population coincided with measures taken by the broiler industry to reduce antimicrobial resistant bacteria
Source: PLoS One. 2019 Dec 12;14(12):e0226101. doi: 10.1371/journal.pone.0226101 (PMC6907784; doi:10.1371/journal.pone.0226101)

**S1 Appendix.**

**Table A. Occurrence of the ATPase and permease genes encoding the ABC-type membrane transporter putatively conferring reduced susceptibility to narasin in the vancomycin resistant *Enterococcus faecium* isolates from broilers included in this study.**

| **Sample** | **Strain** | **Species** | **Results** | | **Comment** | **VAN** | **NAR** |
| --- | --- | --- | --- | --- | --- | --- | --- |
|  |  |  | **ATPase**  **(476 bp)** | **Permease**  **(303 bp)** |  |  |  |
| **1** | 2006-01-1110 | broiler | + | + | VRE | R | RS |
| **2** | 2006-01-1151 | broiler | + | + | VRE | R | RS |
| **3** | 2006-01-1158 | broiler | + | + | VRE | R | RS |
| **4** | 2006-01-1402 | broiler | + | + | VRE | R | RS |
| **5** | 2006-01-2406 | broiler | + | + | VRE | R | RS |
| **6** | 2006-01-2609 | broiler | + | + | VRE | R | RS |
| **7** | 2006-01-3432 | broiler | + | + | VRE | R | RS |
| **8** | 2006-01-3433 | broiler | + | + | VRE | R | RS |
| **9** | 2009-01-1177-2 | broiler | + | + | VRE | R | RS |
| **10** | 2009-01-1357-3 | broiler | + | + | VRE | R | RS |
| **11** | 2009-01-1357-5 | broiler | + | + | VRE | R | RS |
| **12** | 2009-01-1455-2 | broiler | + | + | VRE | R | RS |
| **13** | 2009-01-1808-3 | broiler | + | + | VRE | R | RS |
| **14** | 2009-01-1808-4 | broiler | + | + | VRE | R | RS |
| **15** | 2009-01-1964-1 | broiler | + | + | VRE | R | RS |
| **16** | 2009-01-2138-4 | broiler | + | + | VRE | R | RS |
| **17** | 2009-01-2393-3 | broiler | + | + | VRE | R | RS |
| **18** | 2009-01-2574-2 | broiler | + | + | VRE | R | RS |
| **19** | 2009-01-2574-4 I | broiler | + | + | VRE | R | RS |
| **20** | 2009-01-2807-2 | broiler | + | + | VRE | R | RS |
| **21** | 2009-01-3361-2 | broiler | + | + | VRE | R | RS |
| **22** | 2009-01-3896-4 | broiler | + | + | VRE | R | RS |
| **23** | 2009-01-4415-2 | broiler | + | + | VRE | R | RS |
| **24** | 2009-01-4530-4 | broiler | + | + | VRE | R | RS |
| **25** | 2009-01-5505-5 | broiler | + | + | VRE | R | RS |
| **26** | 2011-01-101-6 I | broiler | + | + | VRE | R | RS |
| **27** | 2011-01-972-1 | broiler | + | + | VRE | R | RS |
| **28** | 2011-01-1544-6 | broiler | + | + | VRE | R | RS |
| **29** | 2011-01-1670-5 | broiler | + | + | VRE | R | RS |
| **30** | 2011-01-2112-1 | broiler | + | + | VRE | R | RS |
| **31** | 2011-01-2301-5 | broiler | + | + | VRE | R | RS |
| **32** | 2011-01-2508-1 | broiler | + | + | VRE | R | RS |
| **33** | 2011-01-2508-6 | broiler | + | + | VRE | R | RS |
| **34** | 2011-01-3460-2 | broiler | + | + | VRE | R | RS |
| **35** | 2011-01-3717-3 | broiler | + | + | VRE | R | RS |
| **36** | 2011-01-3717-5 | broiler | + | + | VRE | R | RS |
| **37** | 2011-01-3991-2 | broiler | + | + | VRE | R | RS |
| **38** | 2011-01-3991-4 | broiler | + | + | VRE | R | RS |
| **39** | 2011-01-4277-6 | broiler | + | + | VRE | R | RS |
| **40** | 2011-01-5250-2 | broiler | + | + | VRE | R | RS |
| **41** | 2011-01-5250-7 | broiler | + | + | VRE | R | RS |
| **42** | 2011-01-5252-1 | broiler | + | + | VRE | R | RS |
| **neg** | Negative control |  | Ok | Ok |  |  |  |
| **pos** | Positive control |  | Ok | Ok |  |  |  |

| **43** | 2011-01-5253-2 | broiler | + | + | VRE | R | RS |
| --- | --- | --- | --- | --- | --- | --- | --- |
| **44** | 2011-01-5253-3 | broiler | + | + | VRE | R | RS |
| **45** | 2011-01-5254-5 | broiler | + | + | VRE | R | RS |
| **46** | 2011-01-5432-1 | broiler | + | + | VRE | R | RS |
| **47** | 2011-01-5610-1 | broiler | + | + | VRE | R | RS |
| **48** | 2011-01-6227-1 | broiler | + | + | VRE | R | RS |
| **49** | 2011-01-6227-6 | broiler | + | + | VRE | R | RS |
| **50** | 2011-01-6462-2 | broiler | + | + | VRE | R | RS |
| **51** | 2011-01-6462-6 | broiler | + | + | VRE | R | RS |
| **52** | 2011-01-6928-3 | broiler | + | + | VRE | R | RS |
| **53** | 2011-01-6928-4 | broiler | + | + | VRE | R | RS |
| **54** | 2011-01-6928-6 | broiler | + | + | VRE | R | RS |
| **55** | 2011-01-7378-4 | broiler | + | + | VRE | R | RS |
| **56** | 2011-01-7660-4 | broiler | + | + | VRE | R | RS |
| **57** | 2011-01-7660-5 | broiler | + | + | VRE | R | RS |
| **58** | 2011-01-7940-5 | broiler | + | + | VRE | R | RS |
| **59** | 2011-01-7940-6 | broiler | + | + | VRE | R | RS |
| **60** | 2011-01-8174-2 | broiler | + | + | VRE | R | RS |
| **61** | 2011-01-8304-2 | broiler | + | + | VRE | R | RS |
| **62** | 2011-01-8304-5 | broiler | + | + | VRE | R | RS |
| **63** | 2011-01-8709-2 | broiler | + | + | VRE | R | RS |
| **64** | 2011-01-9195-3 | broiler | + | + | VRE | R | RS |
| **65** | 2011-01-9386-3 | broiler | + | + | VRE | R | RS |
| **66** | 2014-01-1741 | broiler | + | + | VRE | R | RS |
| **67** | 2014-01-1914 | broiler | + | + | VRE | R | RS |
| **68** | 2014-01-2995 | broiler | + | + | VRE | R | RS |
| **69** | 2014-01-4539 | broiler | + | + | VRE | R | RS |
| **70** | 2014-01-6934 | broiler | + | + | VRE | R | RS |
| **71** | 2014-01-7050 | broiler | + | + | VRE | R | RS |
| **72** | 2014-01-7207 | broiler | + | + | VRE | R | RS |
| **73** | 2014-01-7377 | broiler | + | + | VRE | R | RS |
| **74** | 2014-01-7394 | broiler | + | + | VRE | R | RS |
| **75** | 2014-01-7479 | broiler | + | + | VRE | R | RS |
| **76** | 2014-01-7483 | broiler | + | + | VRE | R | RS |
| **77** | 2014-01-7512 | broiler | + | + | VRE | R | RS |
| **78** | 2014-01-7513 | broiler | + | + | VRE | R | RS |
| **79** | 2014-01-7584 | broiler | + | + | VRE | R | RS |
| **neg** | Negative control |  | Ok | Ok |  |  |  |
| **pos** | Positive control |  | Ok | Ok |  |  |  |

VRE; vancomycin resistant *Enterococcus faecium*, VAN; vancomycin, NAR; narasin, RS; Reduced susceptible

**Figure A. Gel pictures showing PCR products of the ATPase gene of the vancomycin resistant isolates included in this study.**


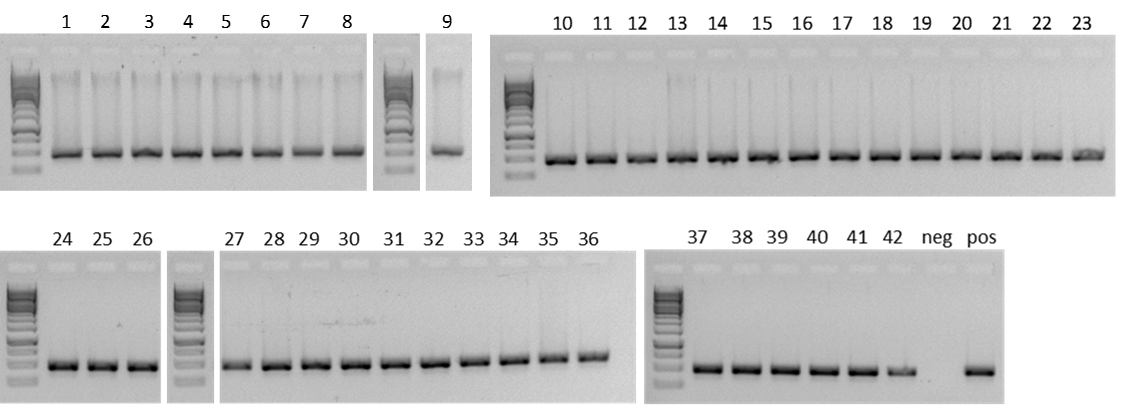


**
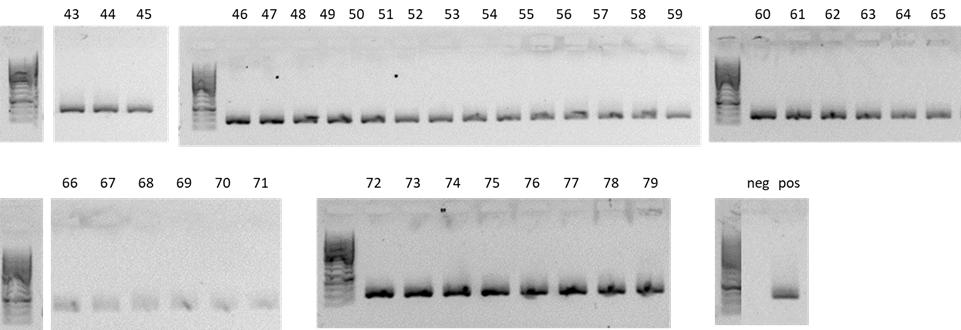
**

**Figure B. Gel pictures showing PCR products of the permease gene of the vancomycin resistant isolates included in this study.**

**
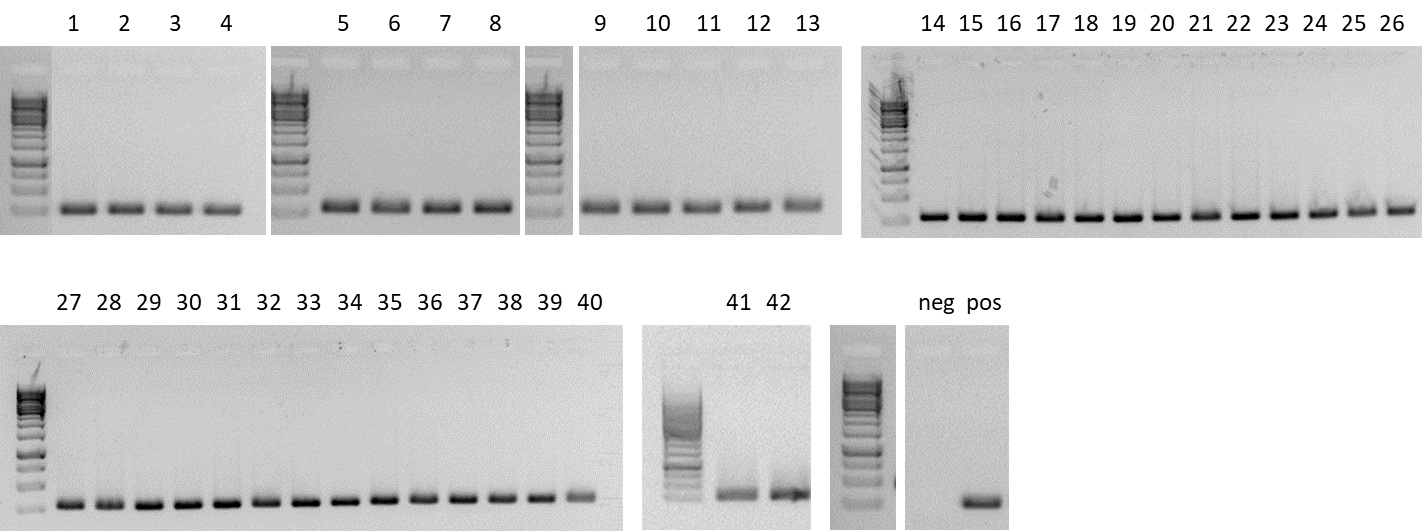
**


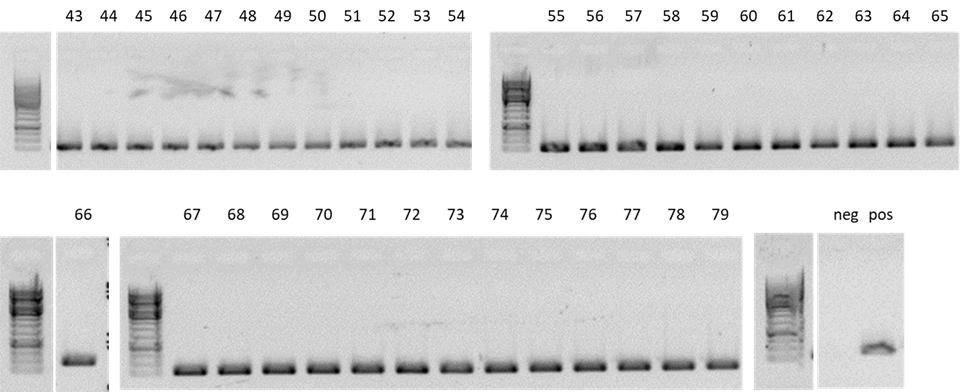

Supplement: S1 Appendix — (DOCX) [file pone.0226101.s001.docx]
