# Supplementary material for: Significant reduction of vancomycin resistant E. faecium in the Norwegian broiler population coincided with measures taken by the broiler industry to reduce antimicrobial resistant bacteria
Source: PLoS One. 2019 Dec 12;14(12):e0226101. doi: 10.1371/journal.pone.0226101 (PMC6907784; doi:10.1371/journal.pone.0226101)
Supplement: S1 Table — (DOCX) [file pone.0226101.s002.docx]

**S1 Table. Isolation methods.**

| **Year** | **Material** | **Method** | **Agar plate** | **Incubation** | **Comment** |
| --- | --- | --- | --- | --- | --- |
| 2000 | Meat | *Enterococcus* spp. | Slanetz and Bartley | 37±1°C; 48h | Enrichment in azide dextrose; 44±1°C; 24h |
| 2002 | Meat | *Enterococcus* spp. | Slanetz and Bartley | 37±1°C; 48h | Enrichment in azide dextrose; 44±1°C; 24h |
|  | Faecal | *Enterococcus* spp. | Slanetz and Bartley | 37±1°C; 48h |  |
|  | Faecal | VRE | Slanetz and Bartley with 32 mg/L vancomycin | 37±1°C; 48h | Serial dilution of sample. |
| 2004 | Meat | *Enterococcus* spp. | Slanetz and Bartley | 37±1°C; 48h | Enrichment in azide dextrose; 44±1°C; 24h |
|  | Faecal | *Enterococcus* spp. | Slanetz and Bartley | 37±1°C; 48h |  |
|  | Faecal | VRE | Slanetz and Bartley with 32 mg/L vancomycin | 37±1°C; 48h |  |
| 2006 | Meat | *Enterococcus* spp. | Slanetz and Bartley | 37±1°C; 48h | Enrichment in azide dextrose; 44±1°C; 24h |
|  | Faecal | *Enterococcus* spp. | Slanetz and Bartley | 37±1°C; 48h |  |
|  | Faecal | VRE | Slanetz and Bartley with 32 mg/L vancomycin | 37±1°C; 48h |  |
| 2009 | Faecal, boot swab | VRE | Slanetz and Bartley with 32 mg/L vancomycin | 44±1°C; 48h |  |
| 2011 | Faecal, boot swab | *Enterococcus* spp. | Slanetz and Bartley | 44±1°C; 48h |  |
|  | Faecal, boot swab | VRE | Slanetz and Bartley with 32 mg/L vancomycin | 44±1°C; 48h |  |
| 2014 | Caecal | *Enterococcus* spp. | Slanetz and Bartley | 41.5±1°C; 48h | Ten animals per sample |
|  | Caecal | VRE | Slanetz and Bartley with 4 mg/L vancomycin | 41.5±1°C; 48h | Ten animals per sample |
| 2018 | Caecal | *Enterococcus* spp. | Slanetz and Bartley | 44±1°C; 48h | Ten animals per sample |
|  | Caecal | VRE | Slanetz and Bartley with 4 mg/L vancomycin | 44±1°C; 48h | Ten animals per sample |
